# Caesarean delivery rate and indications at a secondary healthcare Facility in Ibadan, South Western Nigeria: a five-year review

**DOI:** 10.4314/ahs.v21i1.41

**Published:** 2021-03

**Authors:** Waheed O Ismail, Ibrahim S Bello, Samuel A Olowookere, Azeez O Ibrahim, Tosin A Agbesanwa, Wulaimat A Adekunle

**Affiliations:** 1 Department of Family Medicine, Obafemi Awolowo University Teaching Hospitals Complex, Ile-Ife; 2 Accident and Emergency department, Federal Teaching hospital Ido -Ekiti; 3 Family Medicine Department, Ekiti State University Teaching Hospital, Ado -Ekiti; 4 Centre for Family Medicine, Jericho Specialist Hospital, Jericho, Ibadan

**Keywords:** Caesarean section, rate, secondary healthcare, Nigeria

## Abstract

**Background:**

Caesarean delivery is an essential surgical skill within the primary care setting aimed at reducing maternal morbidity and mortality.

**Objectives:**

To determine the rate and indications for caesarean deliveries with a view to improving on the service delivery in the study area.

**Methods:**

A retrospective review of all caesarean deliveries over a five-year period, January 1^st^, 2012 to December 31^st^, 2016.

**Results:**

A total of 2321 deliveries were recorded during the study duration and 481 of them were through caesarean section (CS) giving a caesarean section rate of 20.4%. The rate was higher in the multigravida 255 (53.1%). The commonest indication for caesarean section was previous caesarean section 131 (27.2%). Emergency caesarean delivery accounted for 278 (57.8%). Only 16 (3.3%) stayed more than five days postoperatively while the rest, 465 (96.7%), stayed less than five days. There was a gradual yearly increase in rate from 12.1% in 2012 to 19.5% in 2016.

**Conclusion:**

The rate of CS in this study has shown a gradual yearly increase with emergency CS having a higher percentage. Early diagnosis and referral of high-risk pregnancies from peripheral hospitals could reduce emergency CS among the study population.

## Introduction

Caesarean delivery (CS) is an essential surgical skill within the primary care setting aimed at reducing maternal morbidity and mortality. It remains the most common major surgery performed on women worldwide^1^,^2^. It is therefore essential to periodically evaluate the reasons for this increase especially in developing countries. World Health Organization (WHO) considers CS rate of 5–15% to be optimal range^4^. Lower rate could suggest unmet needs of the patients while higher rate indicates improper selection at times^2^,^3^. The CS prevalence vary from one country to another with 32% reported in USA, 25% reported in UK, 16–36.4% in China, 25.4% in India, and 35.4% in Latin America^4^–^8^. However, lower rates of 4–16% were reported in some countries in sub-Saharan Africa^3^. In Nigeria, CS rate of 10.2–34.7% has been reported in some teaching hospitals^9^,^10^. The prevalence of CS in Northern Nigeria was 10.1% in Kano, 11.8% in Maiduguri, 17.7% in Jigawa and 19.3% in Makurdi^11^–^13^. Similar findings were reported in Southern Nigeria with 16.6% in Abakaliki, 27.6% in Enugu and 34.5% in Abraka^14^–^16^. Caesarean delivery is indicated in cases where vaginal delivery is either not feasible or imposes undue risks to the mother, the baby or both^2^, ^8^, ^9^. Common indications include prolonged labour, severe pre-eclampsia, Cephalopelvic disproportion, antepartum hemorrhage and fetal distress^9^,^10^,^12^,^17^. Maternal request for CS is an emerging indication^9^, ^12^, ^17^. The common complications associated with CS include postpartum haemorrhage, anemia, endometritis and wound infections^1^, ^4^, ^12^. Administering prophylactic antibiotics and ensuring haemostasis have helped to decrease the incidence of these complications^1^,^4^, ^12^. In view of increasing demand for CS across the world, identifying the rate, indications and outcome of caesarean deliveries in the study area will assist in strengthening planning and formulation of health policy that will minimize morbidity and mortality associated with the procedure. This study assessed the rate and indications for caesarean deliveries (CS) in a secondary healthcare facility in Ibadan, Nigeria.

## Methods

### Study site and Study design

This was a retrospective review of all caesarean deliveries in our facility over a period of five years, from January 1^st^, 2012 to December 31^st^, 2016. The healthcare facility headed by a consultant family physician was supported by other healthcare workers including medical officers, nurses and laboratory scientists. The hospital has 20 beds to care for pregnant women. It serves as a secondary healthcare referral centre for some private hospitals, maternity homes and primary health centres in Oyo State and other neighbouring states. However, very ill pregnant women are referred to the tertiary teaching hospital in Ibadan, Nigeria.

### Data collection

The labour ward and theatre registers provided information on the list of pregnant women attended to during the study period, the number of deliveries and the total number of caesarean deliveries performed over the study duration. The case notes of the women who had caesarean deliveries were retrieved from the records and examined in detail. Information extracted from the case notes included the sociodemographic data of the women: age, marital status, occupation, level of education, social class, age at marriage, parity and booking status. Other information retrieved include indication, type and technique of CS, cadre of the surgeon, type of anaesthesia, fetal and maternal outcomes and associated complications.

### Data analysis

Data were entered into SPSS version 22.0 for windows and analyzed using descriptive statistics. Continuous variables were analyzed using mean and standard deviation (SD) while categorical variables were analyzed using proportions and percentages. Results are presented in tables and figures.

### Ethical consideration

Permission to conduct the study was obtained from the head of the hospital while ethical clearance was granted by the Ethical and Research Committee in the state. Confidentiality of patients' records was maintained. The data collected were entered and kept in a password-protected computer.

## Results

A total of 2321 deliveries were recorded during the study duration and 481 of them were through caesarean section (CS), giving a caesarean section rate of 20.4%. The mean age was 31.57 years (SD: 4.44), in a range of 16–45 years. The age range 30–34 years had the highest percentage of subjects in this study (39.3%). Majority of subjects had tertiary education, 287 (59.7%) and 281 (58.4%) were civil servants. Almost all the pregnant women were in social class I & II, 458 (95.2%). [Table 1].

**Table 1:** Socio-demographic characteristic of patients (n=481)

Variables	Frequency(n)	%
Age (years)		
<19	1	0.2
19–24	20	4.2
25–29	147	30.5
30–34	189	39.3
≥35	124	25.8

Education		

Primary	4	0.8
Secondary	190	39.5
Tertiary	287	59.7

Social class		
Upper class (1 and II)	458	95.2
Others (III, IV and V)	23	4.8

Occupation		
Civil servant	281	58.4
Housewife	3	0.6
Trading	94	19.5
Student	28	5.8
Self-employed	54	11.2
Unemployed	21	4.5

Marital status		
Married	480	99.8
Unmarried	1	0.2

One hundred and thirty-one pregnant women (27.2%) had previous scars as an indication for previous cesarean deliveries while eclampsia constituted 0.4%. [Table 2]

**Table 2:** Indication for Caesarean deliveries

Indications	Frequency (n=481)	%
Previous scars	131	27.2
Fetal distress	82	17.0
Failed induction/reduced	61	12.7
liquor		5.8
Macrosomia	28	5.8
Prolonged obstructed labour	28	5.6
Pregnancy induced hypertension	27	4.2
Twin Gestation with Malpresentation of the Leading twin	20	4.0
Breech Presentation	19	3.5
Maternal request	17	3.3
Elderly Prim Gravida/Primary infertility	16	
Cephalopelvic disproportion	12	2.5
Preeclampsia	10	2.1
Cervical Dystocia	10	2.1
Transverse lie at term	7	1.4
placenta praevia type III	6	1.2
Antepartum haemorrhage	5	1.0
Eclampsia	2	0.4

**Figure 1:**
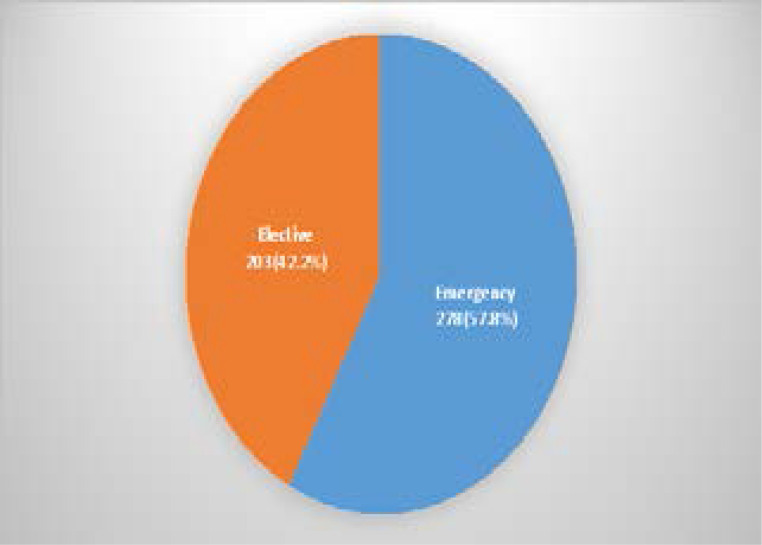
Type of Caesarean Surgery

Two hundred and seventy-eight pregnant women, (57.8%) had emergency cesarean surgery done. [Figure 1]

**Figure 2:**
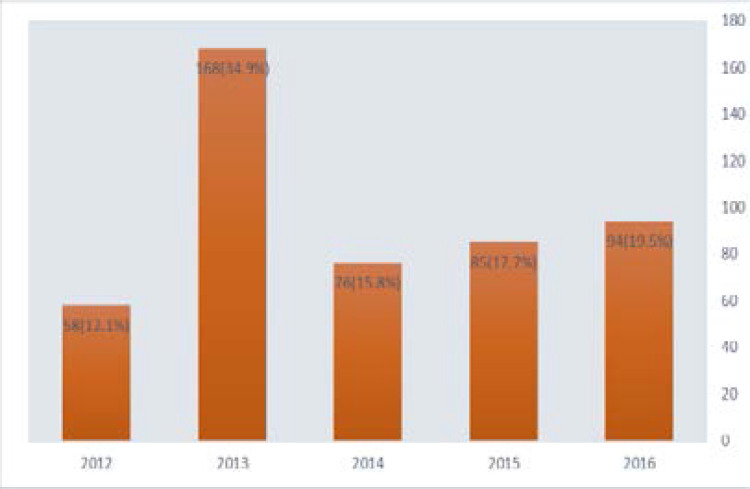
Yearly distribution of Caesarean deliveries rate from 2012 to 2016

There is steady increase in cesarean rate from 12.1% to 19.5% from year 2012 to 2016. [Figure 2]

**Figure 3:**
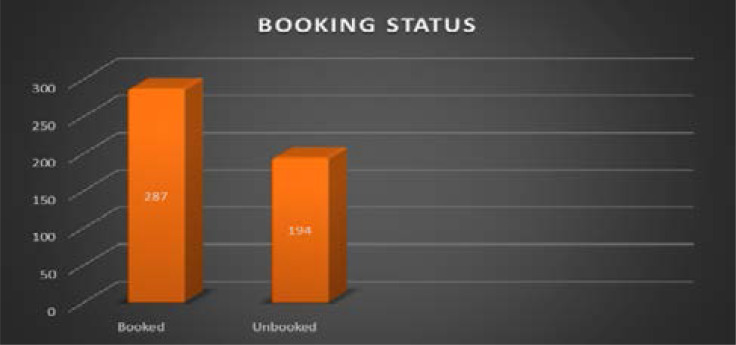
Frequency distribution of booking status

One hundred and ninety-four pregnant women (40.3%) were unbooked. [Figure 3]

Majority of the pregnant women were multigravida, 255 (53.0%). [Table 3]

**Table 3:** Parity distribution of Patients

Parity	Frequency(n=481)	%
0	225	46.8
1–4	255	53.0
≥5	1	0.2

## Discussion

This study assessed the rate and indications for CS in a secondary healthcare facility in Ibadan, Nigeria. The overall CS rate in this study was 20.4% which is higher than the WHO recommended optimal value of 5–15%. However, our finding is consistent with findings of 19.3% and 21.4% in Makurdi and Abuja respectively^9^,^13^ but is higher than 10.1%, 11.8 % and 17.7% obtained from Kano^11^, Maiduguri^10^, and Jigawa^12^. The differences in CS rate may be as a result of the study area serving as a referral hospital for other healthcare facilities within its catchment area. Also, the figure (20.4%) is less than the reported findings of 25% (UK) 6, 32% (USA)^5^ and 35.4% Latin America^4^. The increasing high rate of CS in these countries could result from their small family size and fear of litigation that could follow if CS was not done on time^5^,^6^,^9^.

Furthermore, the yearly review of CS rates in this study showed a gradual increase from 12.1% in 2012 to 19.5% in 2016. Our hospital is an accredited facility for National Health Insurance Scheme (NHIS) providing financial coverage for the patients on the scheme thereby reducing to the bearest minimum the financial burden of CS. Other possible reasons which can be advanced for the steadily increasing caesarean deliveries in our hospital include the location of the hospital in government reserved area which encourages elites to assess the facilities when compared to the stress and financial expenses of the tertiary hospital. The CS rate tends to multiply or double during industrial strike by health workers in tertiary health institutions coupled with referral from other nearby government General Hospital, private clinics and maternity homes and traditional birth attendants.

The majority of caesarean sections in this study (57.8%) were performed as emergencies procedure even though majority of the patients were unbooked (referred to our facility). However, it is not surprising because the commonest indication was previous CS, where some of the patients had failed vaginal birth after CS or previous CS with additional obstetric complication such as hypertensive disorders of pregnancy and postdatism. Some of the previous studies in Maiduguri^10^, Jigawa^12^, Enugu^15^, Abraka^16^ and Sagamu^18^, all in Nigeria, had found emergency CS commonly among the unbooked patients. This could be attributed to the fact that unbooked population are likely to present as emergencies as a last resort, when there are impending complications^12^,^18^. Financial constraint may be responsible for this paradox as only those who can afford the cost will access services in such institution. The leading indications for caesarean section in this study were previous caesarean section, fetal distress and failed induction of labour. They are similar to the reports of previous authors in other centres in Nigeria^10^,^16^,^18^. These findings were also similar in other parts of Sub-Saharan Africa^21^. In contrast, studies from Isan et al^9^, Ugwa et al^12^ and other developing countries reported cephalopelvic disproportion and obstructed labour as the commonest indications. These differences may be due to the older age (25–29 years) of this study's participants whose pelvic would have attained maturity for parturition. Studies have shown that cephalopelvic disproportion is related to the age of women at first pregnancy^9^,^12^. Previous caesarean section accounts for as much as 50–60% of cases of repeat caesarean section^1^,^2^,^10^,^19^. Efforts to lower caesarean section rates should, therefore, include reduction in primary caesarean section rates and attempts at vaginal delivery after caesarean section^19^,^20^.

Prolonged obstructed labour was the commonest morbidity in this study and was responsible for prolonged stay for more than five days. This was similar to the finding of Isan et al^9^. This may be related to the number of unbooked cases and procedure performed as emergency CS^12^,^17^.

Unbooked patients may compromise aseptic procedure, making it imperative for them to receive intravenous prophylactic antibiotics for 72 hours. In this study, 97.1% of caesarean sections were performed by the experienced senior medical officers who have spent more than five years in practice. While those less than five years were with closed supervision of the Consultant. There was no recorded mortality due to CS throughout the study duration due to prompt referral at the point of entry to nearby tertiary health facility or after initial stabilization. This forms part of our limitations to handle severe cases.

No study has been done to assess the rate and indications for CS in our hospital, which explains our decision to conduct this review. This study provided useful information to policymakers on the CS rate and its indications.

## Conclusion

The current rate of caesarean delivery in the hospital is high in comparison to World Health Organization and it depicts a step-wise rise in rate from 2012 to 2016, with an exceptional in 2013 as result of industrial strike in the tertiary institution. CS was common among the pregnant women especially among unbooked, multigravida women with previous scar making emergency CS quite common. Prolonged obstructed labour was the commonest morbidity in this study that was responsible for prolonged stay. It is thus recommended that efforts should be made towards reducing primary caesarean section rate in addition to more encouragement of vaginal delivery after one previous caesarean section. There is need for more public awareness on early booking, regular antenatal clinic attendance and prompt referral of high-risk pregnant women from health facilities around the study area. Improvement on facilities and strengthening of secondary healthcare facility is advocated.
